# Permafrost extent sets drainage density in the Arctic

**DOI:** 10.1073/pnas.2307072120

**Published:** 2024-02-01

**Authors:** Joanmarie Del Vecchio, Marisa C. Palucis, Colin R. Meyer

**Affiliations:** ^a^Department of Earth Sciences, Dartmouth College, Hanover, NH 03755; ^b^Neukom Institute for Computational Science, Dartmouth College, Hanover, NH 03755; ^c^Thayer School of Engineering, Dartmouth College, Hanover, NH 03755

**Keywords:** permafrost, drainage density, hydrology, carbon stocks, geomorphology

## Abstract

Amplified warming of high latitudes and rapid thaw of frozen ground threaten permafrost carbon stocks. The presence of permafrost modulates water infiltration and flow, as well as sediment transport, on soil-mantled slopes, influencing the balance of advective fluvial processes to diffusive processes on hillslopes in ways that are different from temperate settings. These processes that shape permafrost landscapes also impact the carbon stored on soil-mantled hillslopes via temperature, saturation, and slope stability such that carbon stocks and landscape morphometry should be closely linked. We studied >69,000 headwater basins between 25° and 90 °N to determine whether the thermal state of the soil sets the balance between hillslope (diffusive) and fluvial (advective) erosion processes, as evidenced by the density of the channel networks (i.e., drainage density) and the proportion of convex to concave topography (hillslopes and river valleys, respectively). Watersheds within permafrost regions have lower drainage densities than regions without permafrost, regardless of watershed glacial history, mean annual precipitation, and relief. We find evidence that advective fluvial processes are inhibited in permafrost landscapes compared to their temperate counterparts. Frozen soils likely inhibit channel development, and we predict that climate warming will lower incision thresholds to promote growth of the channel network in permafrost landscapes. By demonstrating how the balance of advective versus diffusive processes might shift with future warming, we gain insight into the mechanisms that shift these landscapes from sequestering to exporting carbon.

## Significance

In permafrost landscapes, the competition between channel and hillslope processes directly impacts the amount of stored soil organic carbon. However, conceptual models disagree whether the presence of permafrost (and its subsequent thaw) lengthens or shortens channel networks on hillslopes, complicating predictions of carbon release under landscape disturbance. Our compilation of >69,000 watersheds showed that landscapes underlain by permafrost have fewer channels per watershed area (drainage density) and fewer river valleys compared to their temperate counterparts. Limited channelization likely results from frozen ground, which is vulnerable to climate-induced change. Geomorphically vulnerable hillslope positions may store significant organic carbon, prone to oxidation and greenhouse gas production if exposed; one degree of warming could lead to the equivalent emissions of 35 million cars.

Increasing Arctic air temperatures have led to intensification of the hydrologic cycle, reduction of spring snow cover, and warming of near-surface permafrost (1). Amplified warming at high latitudes disturbs permafrost landscapes and ecosystems, potentially disrupting global carbon fluxes through nascent warming feedback processes and thus threatening global emission goals (2, 3). The precise geomorphic mechanisms operating on permafrost landscapes as they evolve should determine whether they act as carbon sources (4) or sinks (5). However, the unique rheological and hydrological properties of permafrost landscapes that make their evolution sensitive to climate change complicate estimates of sediment and carbon fluxes in the midst of climate warming (6).

The size and shape of hillslopes and rivers elucidate the underlying processes moving sediment and water on a landscape. The spacing of ridges and valleys is set by the competition between diffusive processes (creep-like mass transport that distributes sediment across a hillslope), which smooths the landscape, and advective fluvial processes, which incise the landscape (7). In temperate landscapes, the length, curvature, and relief of hillslopes are a function of the pace and pattern of soil movement, which are controlled in part by climate and ecology (8–10). Topography can also elucidate how water moves through a landscape; the density and areal extent of the river network are set by the forces acting on the soil profile as it receives and transmits water from upslope and from precipitation (11, 12). As with soil transport, climate and ecology can control the formation of a fluvial network by setting the permeability structure and erodibility of soil as well as the overall volume of water transiting the system (11, 13). Drainage density is therefore influenced by “top-down” (climate) and “bottom-up” (geology and soil properties) processes that control when advection (fluvial incision) overcomes diffusion (soil creep).

**Fig. 1.**
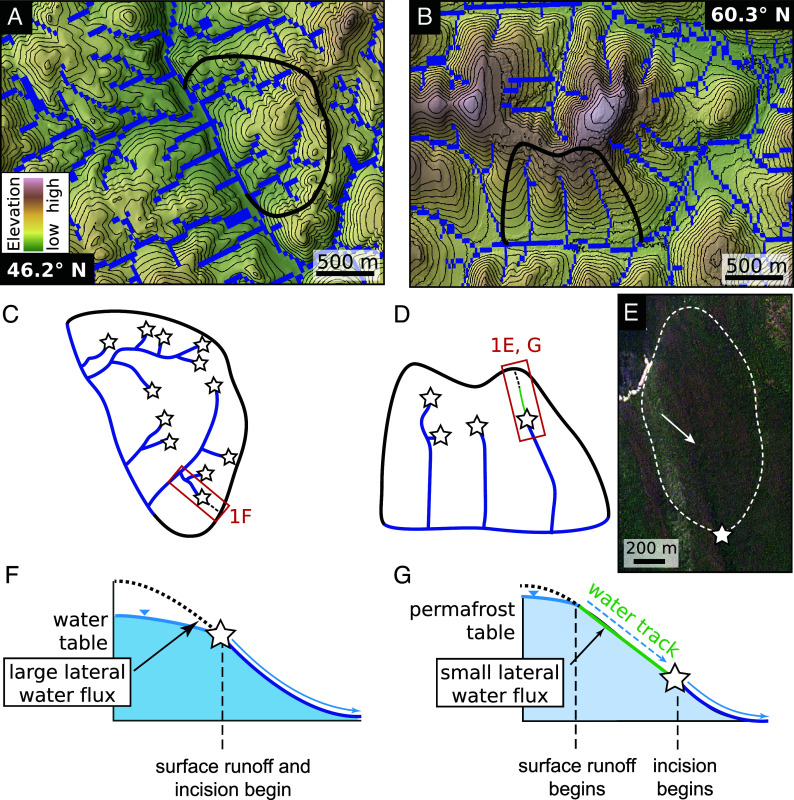
Comparison between drainage densities and hydrogeomorphic configurations in permafrost and temperate landscapes of comparable relief and annual precipitation. (*A*) Hydrography90m stream segment data mapped on a 10 m digital elevation model of a temperate landscape, colored by elevation with 25 m contours. In this landscape about 13% of pixels are occupied by channel segments. (*B*) The same data from a permafrost landscape in which about 10% of pixels are occupied by channel segments, indicating that there are 0% more channelized pixels in the temperate landscape. Black outlines in (*A*) and (*B*) show areas represented in the schematic of drainage networks shown in (*C*) and (*D*), respectively. (*C* and *D*) Schematic of drainage network configurations in map view, where channels are shown as blue lines and the locations of channel heads are shown as stars. (*E*) Orthorectified PlanetScope imagery dated June 2021 of the red box in (*D*). The white arrow points to an area upslope of the channel head (star) in which flowpaths within the dashed white area coalesce to a ∼70 m-wide water track, which experiences greening earlier in the season than the surrounding hillslope. Image © 2021 Planet Labs PBC. (*F*) Cross-section of temperate hillslope demonstrating the co-occurrence of overland flow and channelization. Large lateral water fluxes result from broad upslope watersheds. (*G*) Cross-section of hillslope underlain by permafrost, in which overland flow emerges higher up on the hillslope, but lateral flows and incision within water tracks are limited by frozen ground and vegetation, resulting in narrow upslope watersheds and channelization farther downslope. Red boxes in (*C*) and (*D*) show cross-section details in (*F*) and (*G*).

The presence of permafrost modulates water infiltration, lateral flow, and sediment transport on soil-mantled slopes. In permafrost landscapes, soil transport rates are set by the thermal and saturation state of the soil profile, and sediment flux is thought to be maximized when a permafrost table is present, mean annual temperature is 0 °C, and annual temperature amplitudes are high (14–16). Likewise, tundra vegetation and permafrost soils mediate the flow of water in the subsurface (17). On some frozen hillslopes in the Arctic and Antarctic, the impermeable permafrost table routes surface and subsurface flow paths into zero-order geomorphic features called water tracks (18–22) (Fig. 1). These linear zones of enhanced soil moisture can occur in the absence of well-defined channel valleys (18, 23) because timing of historically peak discharge from winter snow storage and spring melt (24) coincides with minimal ground thaw on hillslopes (18, 23, 24). Previous Arctic studies (18, 25–27) indicated soil-mantled Arctic hillslopes experience relatively limited channel development, but contrasting conceptual frameworks hypothesize that hillslopes underlain by permafrost should exhibit longer channel networks due to the limited capacity for thawed soils to store water (28, 29). This competition between permafrost-modulated erodibility of channels and subsurface water storage, coupled with the importance of thaw-mediated sediment diffusivity, will determine whether permafrost landscapes exhibit a higher density of channels compared to their temperate counterparts.

The same top-down (climate) and bottom-up (geology) processes that control hillslope-channel coupling also influence the spatial variability of soil organic carbon (SOC) (30, 31) such that carbon stocks and landscape morphometry should be closely linked (32). The dominance of diffusive processes over advective ones has the potential to sequester permafrost SOC (5), implying that soil-mantled permafrost landscapes with low drainage densities are likely to act as more efficient carbon sinks than those with more channels. Understanding the influence of climate on drainage density in polar regions allows us to predict the how balance of advective versus diffusive processes will shift with future warming and thus how these landscapes may transition from sequestering to exporting carbon.

Our hypothesis is that, all other factors being equal, a landscape underlain by permafrost will have lower drainage density than a temperate landscape. Advection is limited by frozen ground and diffusion is enhanced by thaw-mediated creep. In order to account for the variety of bottom-up controls on drainage density independent of climate, we sampled >69,000 headwater catchments in the middle and high latitudes of the Northern Hemisphere to determine whether Arctic watersheds had significantly different drainage densities than otherwise-similar temperate watersheds. The large sample size allows us to account for lithologic controls on drainage density in the absence of constraints on substrate properties as well as a variety of tectonic settings.

## Results.

**Fig. 2.**
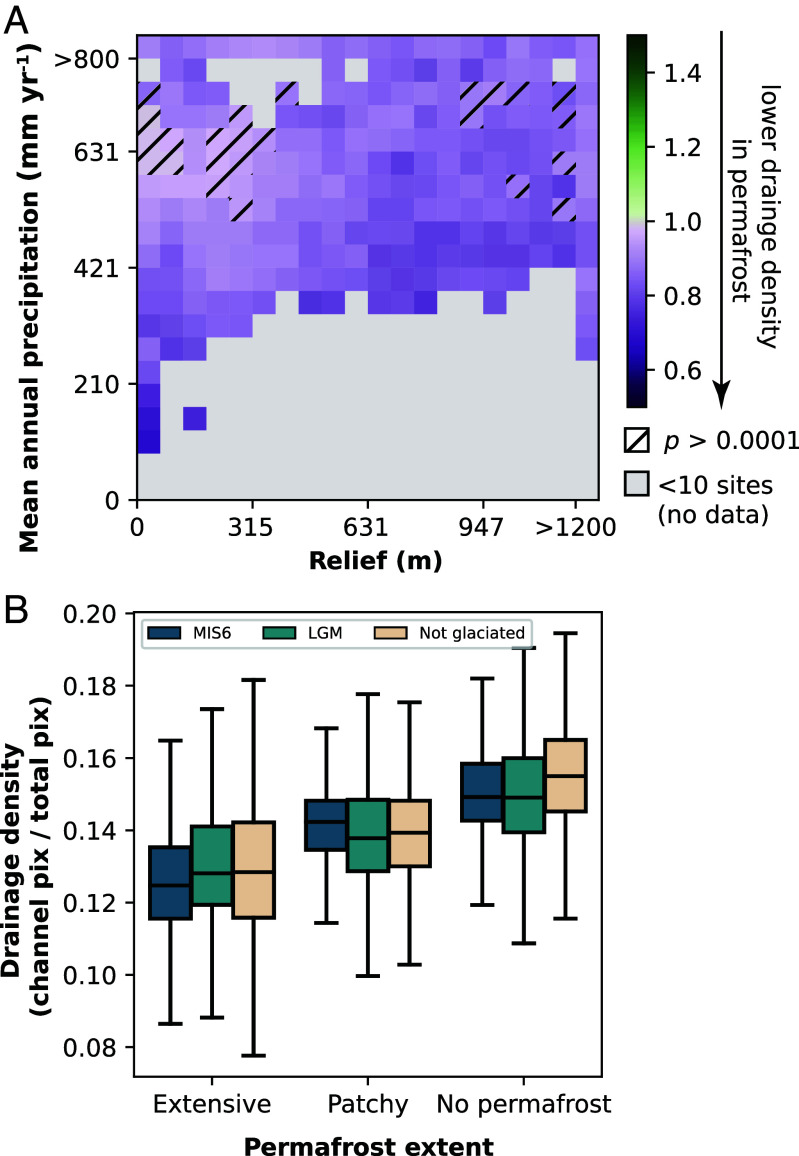
Watershed drainage densities as function of location. (*A*) Ratio of permafrost to non-permafrost drainage density for watersheds binned by relief and mean annual precipitation (MAP). Darker purple values indicate a lower ratio between the two settings (higher drainage density in permafrost watersheds). Hatch marks indicate bins in which a Mann-Whitney *U* test failed below $P=1\times 10^{-4}$ (*Materials and Methods*). Gray data indicate bins where there were fewer than 10 watersheds in either permafrost or non-permafrost settings and thus ratios were not calculated. (*B*) Drainage density as a function of permafrost extent and glacial history. “Extensive” encompasses continuous and discontinuous permafrost, and “patchy” encompasses sporadic and isolated permafrost. “MIS6” is Marine Isotope Stage 6 (191 to 130,000 y ago; “LGM” is Last Glacial Maximum (20,000 y ago).

When binned by relief and mean annual precipitation (MAP), permafrost watersheds have lower drainage densities than their non-permafrost counterparts at a statistically significant level (Fig. 2*A*). This disparity is more pronounced for watersheds with lower MAP, such that arid permafrost watersheds have lower drainage densities than similarly arid unfrozen watersheds. Moreover, the extensiveness of the permafrost impacts drainage density monotonically; continuous and discontinuous permafrost promotes lower drainage density than isolated and sporadic permafrost, and less-extensive permafrost still promotes lower drainage density than landscapes without permafrost (Fig. 2*B*).

We found that this relationship is independent of recent glacial history (Fig. 2*B*), which would otherwise be a primary confounding variable considering the co-location of modern permafrost and ancient ice sheets in the Northern Hemisphere. Instead, for a given glacial history, more extensive permafrost consistently exhibits lower drainage density.

**Fig. 3.**
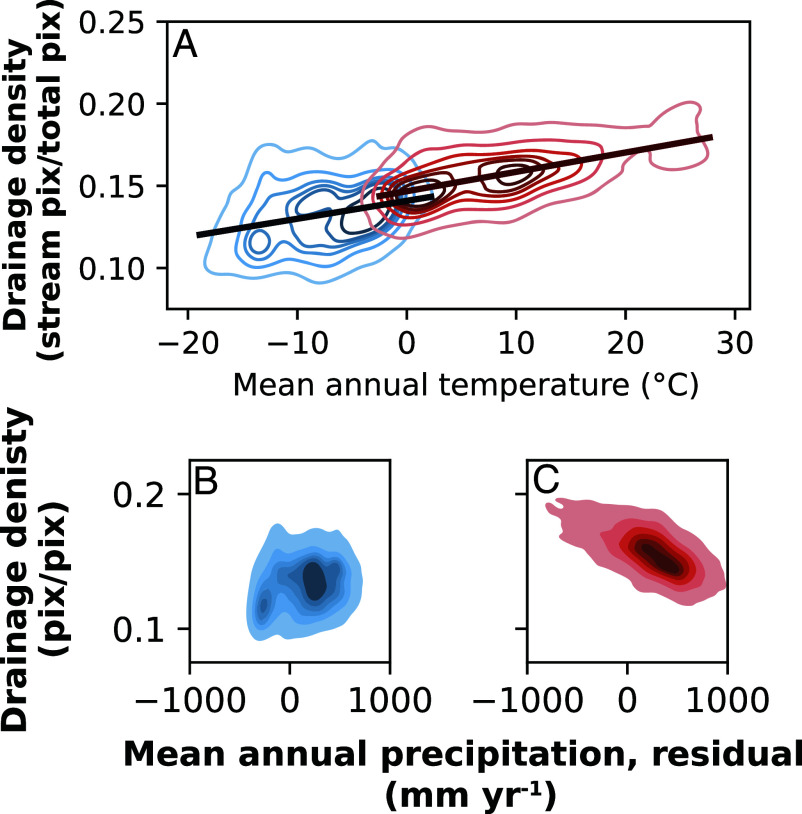
Drainage density of studied watersheds as a function of their mean annual temperature (MAT) and mean annual precipitation (MAP). (*A*) Kernel density estimate plot for visualizing the distribution of drainage density for permafrost watersheds with MAT <2.5 °C (shown in blue) and non-permafrost watersheds with MAT >2.5 °C (shown in red). Linear regression fits are performed separately on the two datasets. (*B* and *C*) An ordinary least squares regression between MAT and MAP was performed to assign each watershed a residual MAP (*SI Appendix*). KDE plots show density of residual values in permafrost (*B*) and non-permafrost (*C*) data with darker colors corresponding to high data density. Residual values for non-permafrost watersheds have a stronger relationship with drainage density, implying that variations in annual precipitation exert some control on drainage density for non-permafrost watersheds, but the relationship is weaker in permafrost watersheds.

We investigated whether there is a correlation between mean annual temperature (MAT) and drainage density, as MAT exerts strong control on the thermal state of the near surface, which we hypothesize sets erodibility of the surface. Higher MATs are associated with higher drainage densities across all headwater catchments we studied regardless of permafrost presence (Fig. 3). MAT is closely related to MAP in our arid and semi-arid sites, especially in the Arctic where higher MATs are generally associated with higher MAPs. To control for the covariation between MAT and MAP, we created a linear regression between MAT and MAP to calculate residual values for MAP. For watersheds not underlain by permafrost, particularly wet watersheds exhibit lower drainage densities than particularly dry watersheds; this trend is opposite and less pronounced for watersheds underlain by permafrost.

**Fig. 4.**
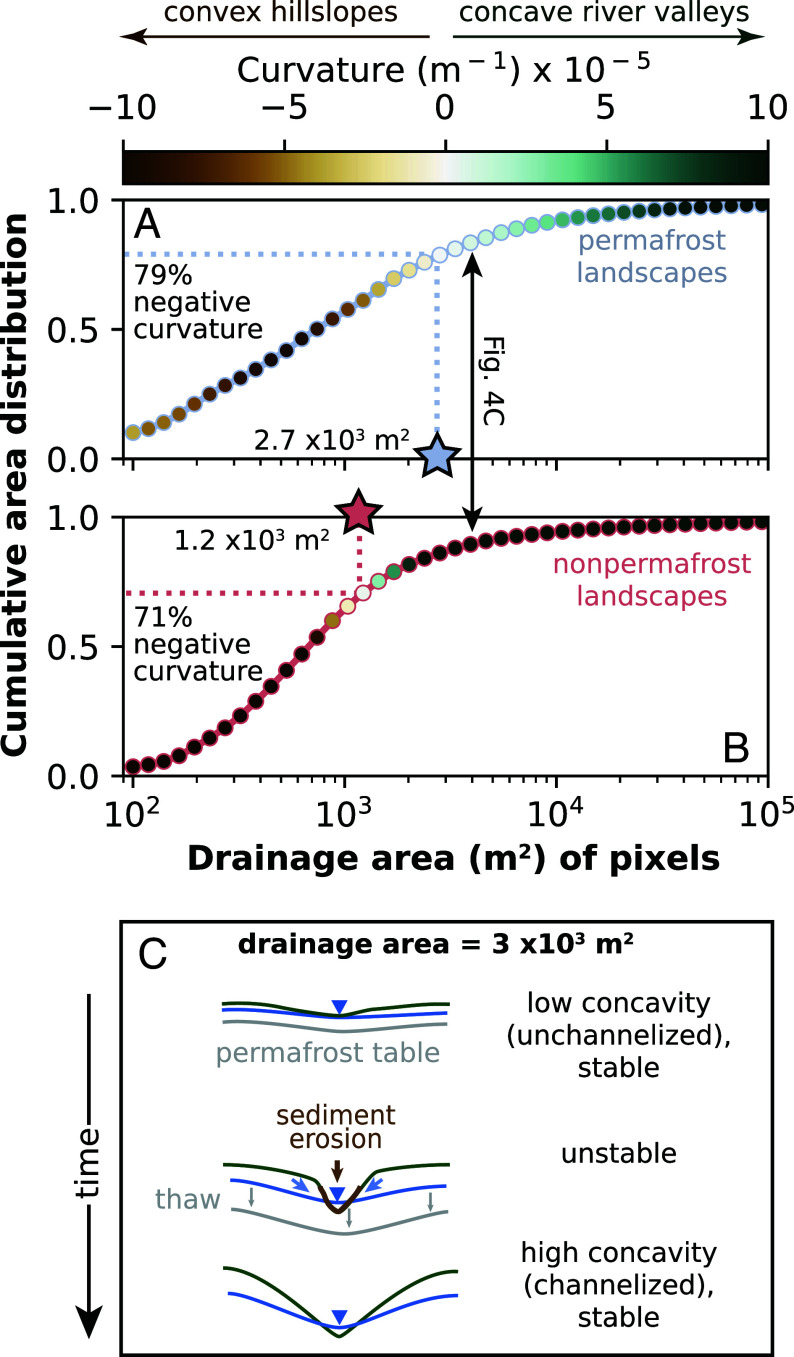
Relationship between curvature, drainage area, and the use of permafrost and non-permafrost landscapes as a space-for-time substitution for the warming Arctic. (*A* and *B*) Median cumulative area distribution and curvatures as a function of drainage area in continuous permafrost (*A*) versus non-permafrost (*B*) landscapes. Stars indicate drainage areas associated with zero median curvature. The black arrow indicates drainage area and curvatures associated with cartoon in Fig. 4*C*. (*C*) Hypothetical evolution of permafrost landscape undergoing warming. Under steady cold conditions, water tracks provide unchannelized flowpaths on permafrost hillslopes, resulting in minimal hillslope convexities. With warming, water tracks coalesce as permafrost tables drop, leading to a positive feedback loop that concentrates subsurface flow, increases flow depth, drives incision of channels, and carves high-convexity valleys while draining nearby water tracks, resulting in erosion and carbon release from soil. In the absence of permafrost, the system stabilizes with river valleys.

Landscape metrics derived from high-resolution topographic data from 476 non-permafrost landscapes and 460 continuous permafrost landscapes corroborate these trends (Fig. 4 *A* and *B*). Permafrost watersheds are characterized by a regime in which intermediate flow accumulation ($10^{3}$ to $10^{4}$ m^2^) occurs on planar to low-curvature slopes; this regime is absent from temperate watersheds, where positions in the landscape with these drainage areas are characterized by relatively high curvature values (>10^−4^ m^−1^). Pixels in permafrost watersheds tend to exhibit more negative curvature values than in non-permafrost watersheds. Valleys form in watersheds underlain by permafrost at higher flow accumulations than in non-permafrost watersheds: The median drainage area for curvatures of $10^{-4}$ m^−1^ is over an order of magnitude higher in permafrost landscapes (46 ×$10^{3}$ m^2^) than in non-permaforst landscapes (2.1 ×$10^{3}$ m^2^).

## Previous Findings Relating Drainage Density to Climate.

Abrahams and Ponczynski (33) observed that drainage density varied inversely with precipitation-evaporation ratios in semi-arid regions while it increased with increasing precipitation-evaporation ratios in humid environments, consistent with earlier work in the American West by Melton (34). This dynamic, in which climate zone dictates the relationship between MAP and drainage density, was studied in numerical simulations (13, 35) and observed in high-resolution topography (36). Using data from sites in the mid-latitude United States, Sangireddy et al. (36) found that, in dry landscapes (<1,050 mm/y precipitation), drainage density decreases with increasing MAP and weakly decreases with increasing relief. Sangireddy et al. (36) also found that increasing vegetation cover results in decreased drainage density. Our study basins, which were filtered for a minimum vegetation index value, fall within the arid and semi-arid classification of the Sangireddy et al.’s study and thus allow us to isolate the effects of temperature and precipitation on drainage density. Although we calculate drainage density on a coarser scale (and show the concordance of our method with traditional drainage density calculations; see *Materials and Methods*), our results show similar relationships for non-permafrost landscapes in which watersheds with higher MAP residuals exhibit lower drainage densities. In contrast, we find that permafrost watersheds exhibit increasing drainage density with increasing MAP despite falling within the arid and semi-arid precipitation classification, implying that different processes control the impact of rainfall on drainage density in the presence of frozen ground.

## Proposed Mechanism.

Arctic vegetation and the impermeable permafrost table mediate the flow of water on the surface and shallow subsurface, notably in water tracks (17, 19). We propose that the historical timing of peak runoff versus thaw conditions (24, 37), paired with tundra ecogeomorphology (21, 38), results in few low-order valleys carved into watersheds underlain by frozen ground, leading to the low drainage densities observed in basins in more extensive permafrost (Fig. 1). Water tracks emerge at the surface or near-surface at hillslope locations with sufficient upslope water (snowmelt, rainfall, or thawed ground ice) to promote the coalescence of flowpaths slightly inset into the background hillslope surface but still underlain by permafrost. At sufficiently large upslope drainage areas ($>10^{4}$ m^2^), these flowpaths have enough thermal and mechanical erosive energy to produce longer-wavelength changes in surface topography and the shape of the permafrost table. These water tracks occupy the topographic space in between a saturation and incision threshold (12), the latter of which is controlled by the thermal state of the soil and the root strength of vegetation. The density of water tracks across a hillslope is likely controlled by constraints on lateral flowpaths into tracks (39), thermal subsidence, and surface processes.

Compared to their temperate counterparts with similar annual precipitation, watersheds with water tracks have many more distributed flowpaths that require a relatively high volume of water to do the geomorphic work of carving valleys that would promote subsequent advective processes. In temperate landscapes, drainage density decreases with higher precipitation, though to be controlled by armoring vegetation (Fig. 3 and (36)). Because we observe the opposite trend in our data, any decrease in erodibility imparted by an increase in vegetation density (13) must be outcompeted by the effect of higher volumes of water in permafrost landscapes.

The proposed mechanism is consistent with field (40, 41) and remote-sensing observations (27) of surface displacement on hillslopes with water tracks. Increased temperatures and rainfall can also generate active-layer detachments in convergent zones on hillslopes (42), an advective process that can drive higher sediment discharges for decades by creating new incised, contiguous channels (43). Although channel expansion via ice wedge degradation (44, 45) does not require convergent topography, tunneling of subsurface flow likely contributes to ground ice thaw- and saturation-driven channel growth across the Arctic (46).

## Warming in Polar Regions and Implications for Carbon Flux.

The negative relationship between permafrost extent and drainage density forecasts the landscape changes that may accompany permafrost thaw under a warming climate. Previous temperature and precipitation conditions maintained frozen soil during peak snowmelt, inhibited incision of permafrost hillslopes. In the future, less precipitation will fall as snow and more precipitation will fall as rain across the Arctic (47, 48). Areas of hillslopes occupied by water tracks are likely to be geomorphically dynamic in the future; deeper and earlier thaw, combined with more rain and less snow, may cause sustained subsidence of the ground under water tracks (27, 38). Inter-track water tables would respond by falling, potentially sapping shallower water tracks of interflow and lead flowpaths to coalesce (Fig. 4*C*). Coalescing water tracks would lead to deeper flows and thus further promote incision into hillslopes and expansion of channel networks.

The CO_2_ flux from tundra surface waters is a product of both erosion of particulate carbon in soils as well as groundwater transport of dissolved carbon (49). Undisturbed, both headwater channels and water tracks can annually emit greenhouse gases important for overall carbon fluxes, but channelized, turbulent flows likely facilitate CO_2_ outgassing and hinder organic matter accumulation compared to water tracks (21, 50, 51). Earlier and deeper thaws, occurring simultaneously with peak flow, not only enable water to incise into mineral soil but also facilitate the maximum leaching of mineral-associated carbon within organic horizons (52). The net carbon loss from this process would be amplified if deeper thaw liberates old carbon for dissolution, transport, and photomineralization (53, 54). We estimate that for every degree of increased MAT, channel area in permafrost watersheds increases by 44,000 m^2^. Based on circumpolar carbon stock estimates to 100 cm depth (55), 4.35 × 10^7^ Gt C are contained within these per-degree areas vulnerable to channel expansion within the studied watersheds. While carbon may accumulate on landscapes for hundreds or thousands of years (5), permafrost channel heads have been observed to migrate tens of meters per year (41, 44), but quantifying these processes’ long-term impact on carbon fluxes has been limited. Post-glacial warming at the beginning of the Holocene (11,650 calendar years before present) denuded Arctic landscapes of sediment (56) and carbon (57) on centennial timescales. Oxidation of carbon derived from permafrost may explain some of the warming at the last deglaciation over ∼2,000 y (58). Thus rapid warming and thaw may reverse thousands of years of soil carbon burial and drive net carbon losses from surface waters, leading to further warming.

## Materials and Methods

### Data Collection.

We used the WWF HydroSHEDS Level 10 Basins dataset (59), a collection of watershed geometries hosted on Google Earth Engine to locate all headwater basins (defined as having no upstream contributing basin) between 23.5° and 90 °N latitude. We further filtered this dataset to find soil-mantled watersheds by only selecting watersheds with average annual normalized difference vegetation index (NDVI) > 0.60, using each pixel’s annual maximum NDVI for the year 2021 as measured by MODIS’s 250 m Terra NDVI product (MOD13A1 v061). We masked the NDVI product by the MODIS water mask product (MOD44W v006). For remaining basins, we extracted mean annual temperature (MAT) and mean annual precipitation (MAP) as reported by WorldClim BIO variables v1 (60), covering data between 1960 and 1991. Because most Arctic watersheds had MAP < 1,000 mm/y, and MAP <1,050 mm/y was found to be the boundary of the inflection point in Sangireddy et al. (36), we eliminated watersheds with >1,000 mm/y precipitation. We calculated basin relief by subtracting the highest and lowest elevations within the watershed of digital elevation models created by NASA’s 90-m resolution Shuttle Ray Topography Mission (SRTM) for 25^°^ to 60^°^ (its upper latitude limit) and 2-m resolution ArcticDEM for 60^°^ to 90^°^ (61). We calculated drainage density using the Hydrography90m dataset (62), which was created using the MERIT Hydro hydrologically conditioned digital elevation model which uses the SRTM dataset with a resolution of ∼90 m at the equator below 60^°^ latitude and the JAXA ALOS 30 m DEM from 60 to 90^°^ latitude (63) to derive a global network of 726 million stream segments with a minimum upstream contributing area of 0.05 km^2^. We masked this dataset with the MODIS water mask and calculated drainage density as the proportion of pixels associated with stream segments to the total number of flow accumulation pixels in the watershed boundary. This method of drainage density calculation is used to report data from Figs. 2 and 3. Once watershed attributes were determined from climate and satellite data, we intersected the centroids of watersheds with the Circum-Arctic Map of Permafrost and Ground Ice Conditions (64) to classify watersheds into categories of continuous, discontinuous, sporadic, isolated, and no permafrost.

### Overall Ratios.

We divided our data into “permafrost” (*n* = 31,974) and “no permafrost” (*n* = 37,618) based on the ground ice extent map. We binned each watershed of each group into one of 20 bins for MAP (range 0 to 1,000 mm/y) and relief (range 0 to 1,200 m) for a total of 400 bins. For bins that contained at least 10 watersheds in each of the permafrost and no permafrost groups, we calculated the ratio of the mean drainage density of permafrost versus non-permafrost datasets for each data bin (lower drainage density in permafrost watersheds results in a ratio <1.0) (Fig. 2*A*). To test the significance of the difference in ratios for each bin, we performed a Mann-Whitney *U* test using the Python package scipy (65) on the distributions of drainage density for permafrost and non-permafrost watersheds. The Mann-Whitney *U* test is a non-parametric test that does not presume a normal distribution between two populations and tests whether any sample of one population will be larger than any sample from the other population. We chose a threshold of $P<10^{-4}$ and differentiate bins that did not pass this significance test with hatches.

### Control of Glacial History.

To confirm that glacial history is not a confounding variable in our study, we intersected the centroids with a map of the extent of the Last Glacial Maximum (LGM) at ∼24 ka at MIS 6 at ∼190 ka (66) to determine whether watersheds of different permafrost extents fell within the LGM boundary (which we assumed also included the MIS 6 extent), the MIS 6 boundary only, or unglaciated. We also performed Mann-Whitney *U* tests on boxplot pairs between permafrost extent categories for each of the glacial histories; all distribution differences are significantly different below a threshold of $p<10^{-4}$ (Fig. 2*B*).

### Control of Mean Annual Temperature.

We selected unglaciated watersheds in our dataset and separated those unglaciated sites into watersheds with (*n* = 16,002) and without (*n* = 23,220) permafrost. We eliminated permafrost watersheds with MAT > 2.5 °C and non-permafrost watersheds with MAT < −2.5 °C. We performed an ordinary least squares (OLS) regression between MAT and drainage density for each group of watersheds and found that generally, drainage density increases with MAT (Fig. 3*A*). To determine the role that MAP might play in the MAT-drainage density relationship (as temperature and precipitation covary), we performed an OLS regression between MAT and MAP for both datasets using the Python package statsmodel and calculated the difference between a site’s actual MAP and the regression fit prediction (residual) for each watershed. This procedure highlighted watersheds with particularly high (“wet”) or low (“dry”) MAP given their MAT. We then regressed these precipitation residuals against drainage density (Fig. 3 *B* and *C*).

### High-Resolution Subset and Comparison to Other Methods.

Both the coarse resolution of the underlying MERIT Hydro digital elevation model (90 m) and the simplified approach to delineating channels by area thresholds are drawbacks to the use of Hydrography90m to calculate drainage density (62). To check whether our results are influenced by either of these factors, we randomly sampled all continuous permafrost and non-permafrost watersheds to generate a subset of unique non-permafrost (*n* = 476) and continuous permafrost (*n* = 460) watersheds that spanned the range of relief and MAP represented by the larger dataset (*SI Appendix*, Fig. S8). We limited the non-permafrost watersheds to the conterminous United States in order to use the 10-m USGS 3DEP National Map Seamless (1/3 Arc-Second) dataset. Of the 936 subset watersheds from continuous permafrost and non-permafrost landscapes, 504 had significant overlap in MAP-relief space and were therefore binned by MAP and relief as in Fig. 2*A*. We downloaded topographic data for each watershed boundary from the USGS dataset (non-permafrost) and used bilinear resampling to downscale the 2-m ArcticDEM dataset to 10 m to match the National Map dataset for continuous permafrost watershed boundaries. We then used the LSDTopoTools software suite (67) to preprocess the DEMs (remove invalid data, fill sinks, and remove dams) and calculate basic topographic metrics (see *SI Appendix* for algorithm parameters). We created flow accumulation rasters with the d-infinity algorithm (68) and calculated tangential curvature with a window of 100 m. We then used the DrEICH algorithm (69), which uses curvature and channel steepness thresholds to select channel heads, to create channel networks using constants $A_{0}$ = 1.0 and m/n = 0.5. We calculated drainage density with the same pixel-counting method as the Hydrography90m dataset and found that while watersheds calculated with the Hydrography90m dataset contained 7× the proportion of channel pixels as the DrEICH algorithm channels (likely due to the difference in pixel resolution of the two datasets), the correspondence between the two drainage density metrics was generally good ($r^{2}$ from OLS regression=0.80; see *SI Appendix*, Fig. S7). The mean of the residual values for this regression is <0 in permafrost and >0 in non-permafrost, meaning that on average, Hydrography90m overestimates permafrost drainage density compared to the DrEICH algorithm and underestimates drainage density in non-permafrost watersheds. We then performed the same comparison between non-permafrost and permafrost drainage densities binned by relief and MAP as described in the text and shown in Fig. 2*A* and found all bins to exhibit lower drainage densities in continuous permafrost watersheds, with most bins exhibiting *P* values < 0.005 (*SI Appendix*, Fig. S8). Despite shortcomings in both channel network delineations given poor remote sensing coverage in polar regions, these independent methods yield the same result.

We used the topographic metrics calculated from the high-resolution subset to asses the dominance of concavity (created by advection) and convexity (created by diffusion) across the landscape independent of channel delineation (Fig. 4 *A* and *B*). For each watershed in the subsample, we calculated a cumulative area distribution for flow accumulation (drainage area) binned by drainage areas of $10^{2}$ to $10^{5}$ m^2^ to determine the relative proportion of flow accumulation values in a watershed. We also found the median tangential curvature for each of these flow accumulation bins to determine the drainage area associated with a switch from convex hillslopes (negative tangential curvature) to concave river valleys (positive tangential curvature). For each type of permafrost extent, we calculated the median cumulative area distribution and tangential curvature for each drainage area bin.

### Carbon Content of Vulnerable Areas.

For permafrost watersheds, we regressed MAT against drainage density (as done in Fig. 3*A*) to calculate that for every degree of higher MAT, a watershed’s channelized pixel percentage (the unit of drainage density used in this study) grows ∼0.15%. We then calculated this area in m^2^ for each watershed (dataset mean= 44,394 m^2^). We assigned each watershed an average carbon mass in the upper 100 cm by intersecting the watershed centroids with the Northern Circumpolar Soil Carbon Database version 2 (55). We multiplied this carbon mass by the per-degree channel expansion area for each watershed to calculate a summed total of 4.35 ×$10^{10}$ kg C. The equivalent mass of CO_2_ equals about 34.6 million passenger cars driving for a year, according to the US EPA (70).

## Supplementary Material

Appendix 01 (PDF)

## Data Availability

All data used in this study are freely available from their original sources, and all codes used to analyze and visualize data can be found at https://doi.org/10.5281/zenodo.7884726 (71), including a .csv data file of watershed analyses. All other data are included in the article and/or *SI Appendix*.
