# Supplementary material for: Permafrost extent sets drainage density in the Arctic
Source: Proc Natl Acad Sci U S A. 2024 Feb 1;121(6):e2307072120. doi: 10.1073/pnas.2307072120 (PMC10861896; doi:10.1073/pnas.2307072120)
Supplement: Supplementary file 1 — Appendix 01 (PDF) [file pnas.2307072120.sapp.pdf]

Supporting Information for

Permafrost extent sets drainage density in the Arctic

Joanmarie Del Vecchio, Marisa Palucis, Colin Meyer

Joanmarie Del Vecchio.
E-mail: joanmarie@dartmouth.edu

This PDF file includes:

Figs. S1 to S11
Tables S1 to S2
SI References

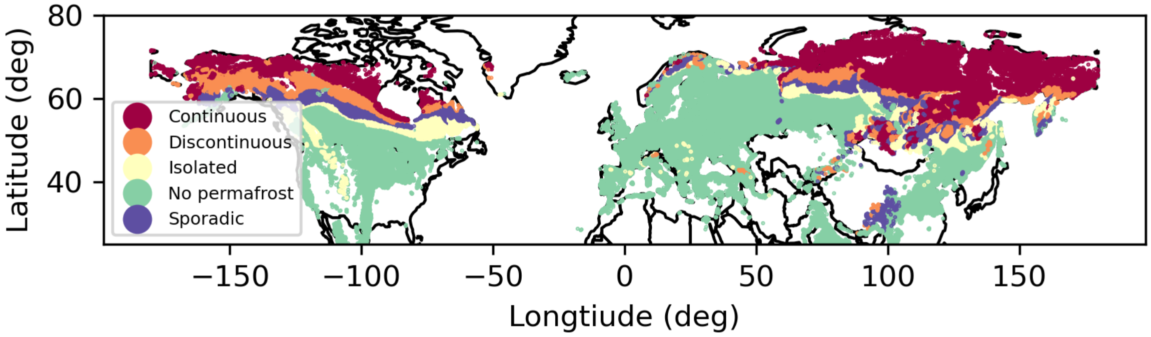

Fig. S1. Permafrost extent of watersheds in study (1)

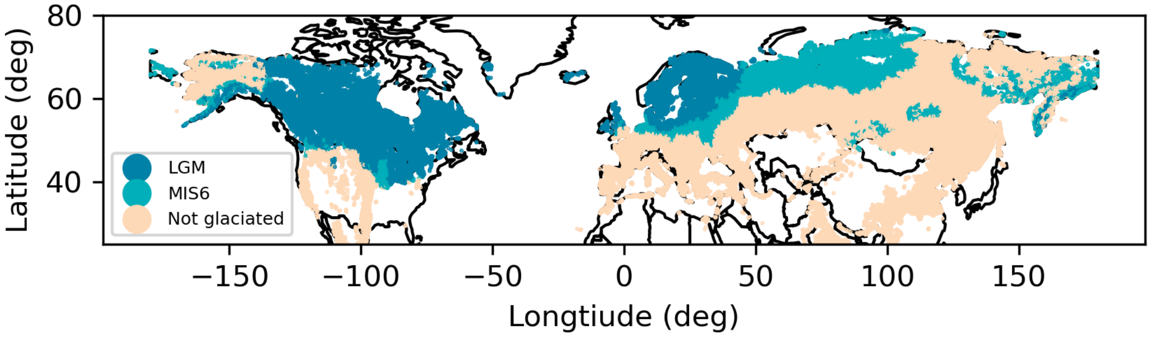

Fig. S2. Glacial history of watersheds in study (2)

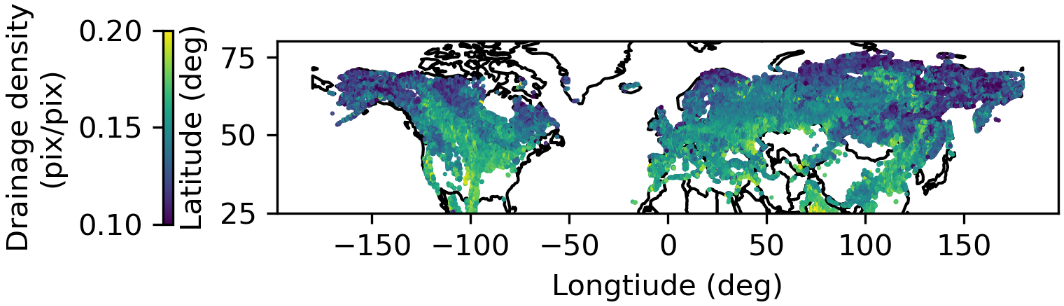

Fig. S3. Drainage density of watersheds in study

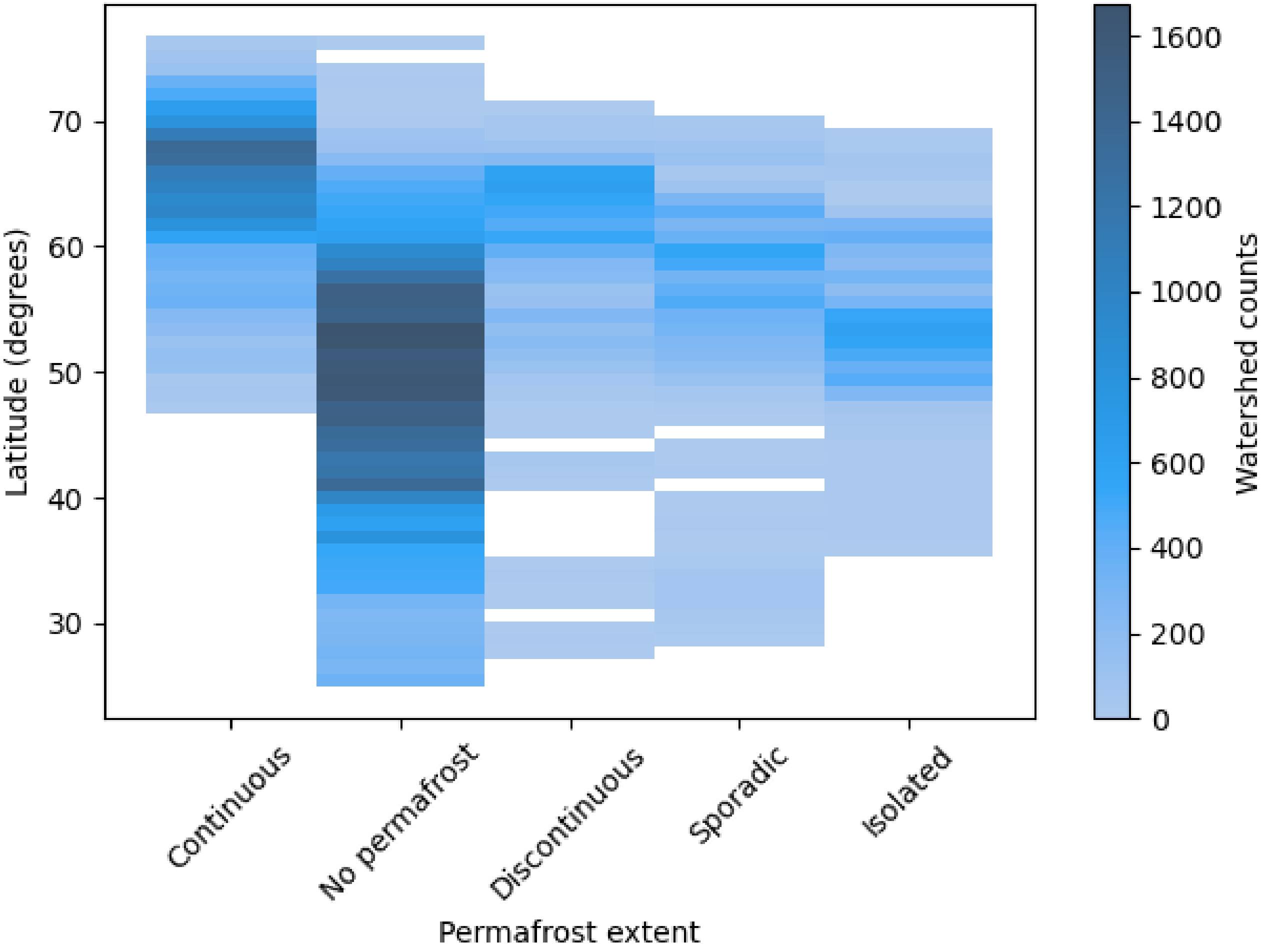

Fig. S4. Number of watersheds in each permafrost extent category by latitud, demonstrating latitude distributions for each category

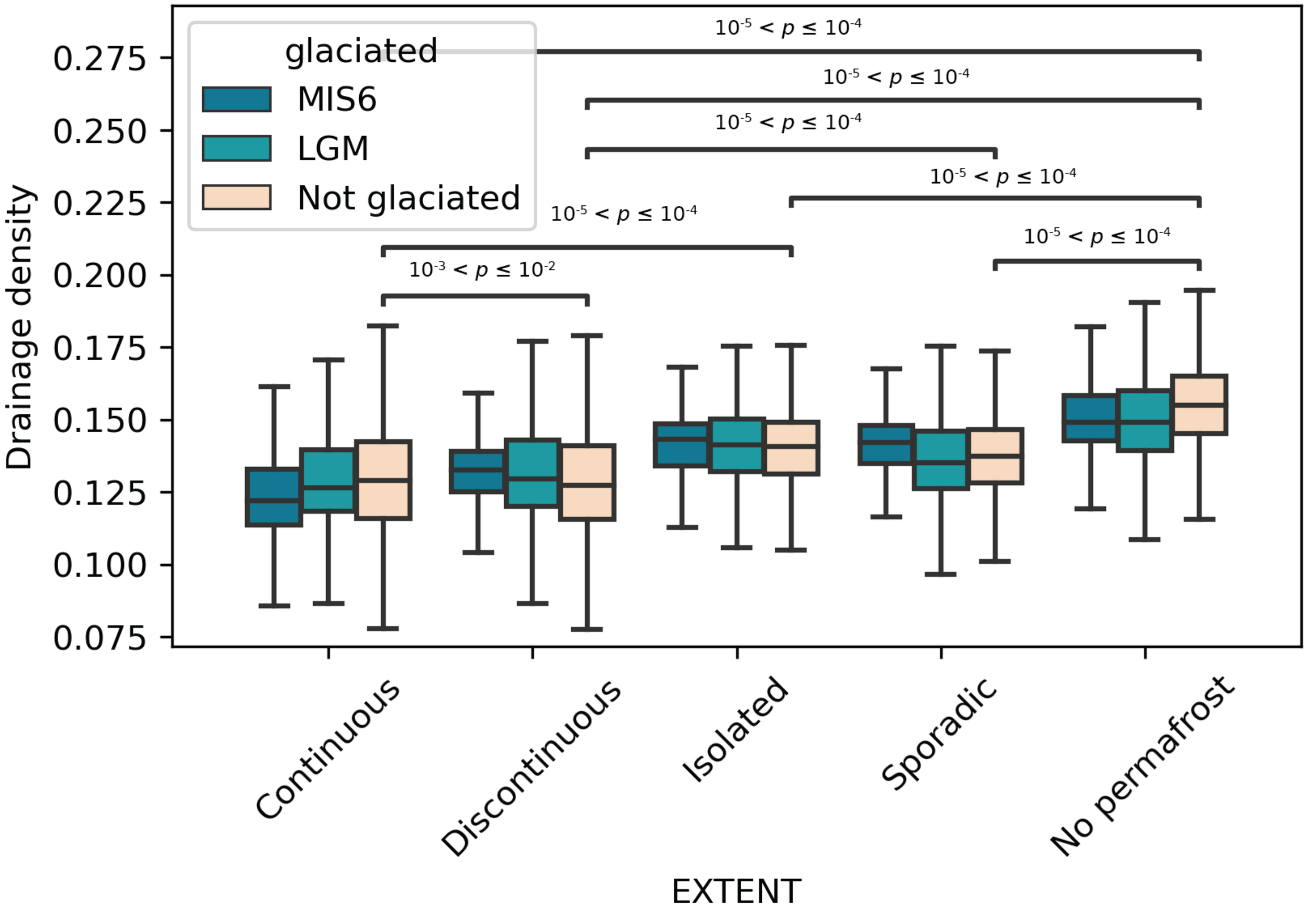

Fig. S5. Boxplot (expanded to five permafrost categories o data from Figure 2B) demonstrating distribution of drainage densities grouped by glacial history and permafrost extent types. The p-value associated with a bracket demonstrates the significance of the associated differences in drainage density calculated from a Mann-Whitney U test.

Joanmarie Del Vecchio, Marisa Palucis, Colin Meyer

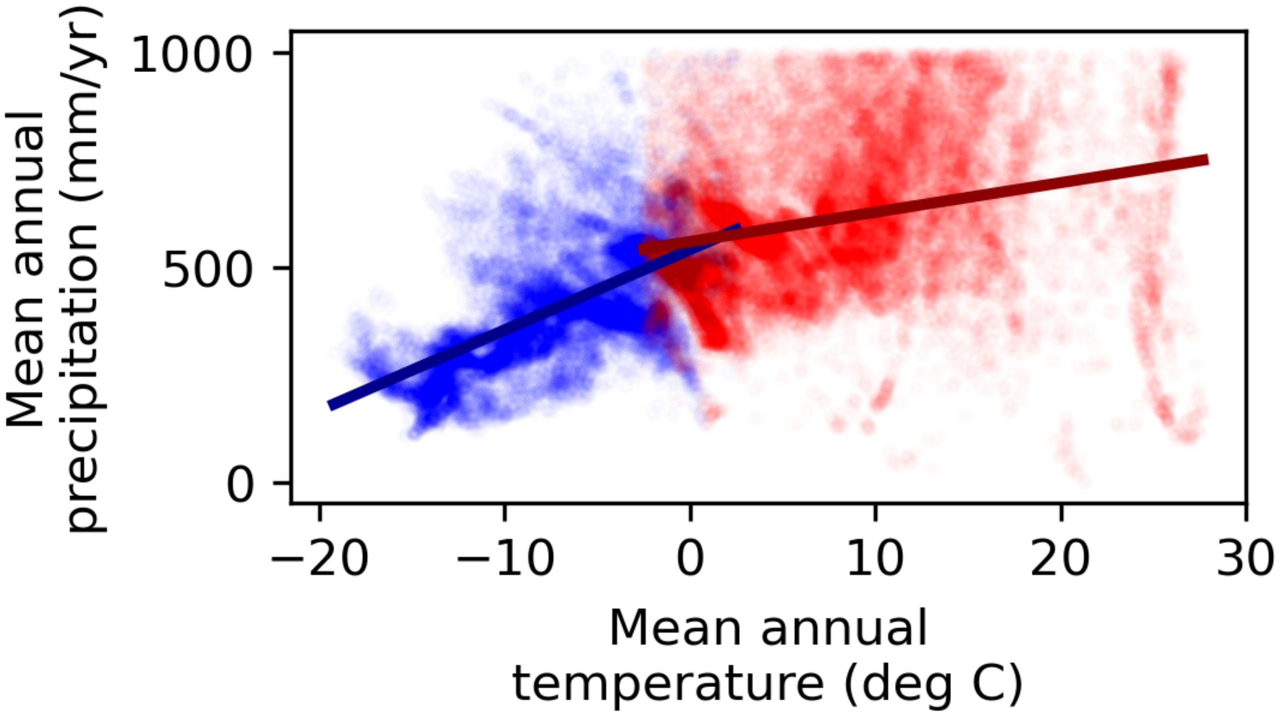

Fig. S6. Regression between mean annual temperature (MAT) and mean annual precipitation (MAP) (3) for permafrost (blue) and non-permafrost (red) watersheds used to calculate residuals for Figure 3B and C.

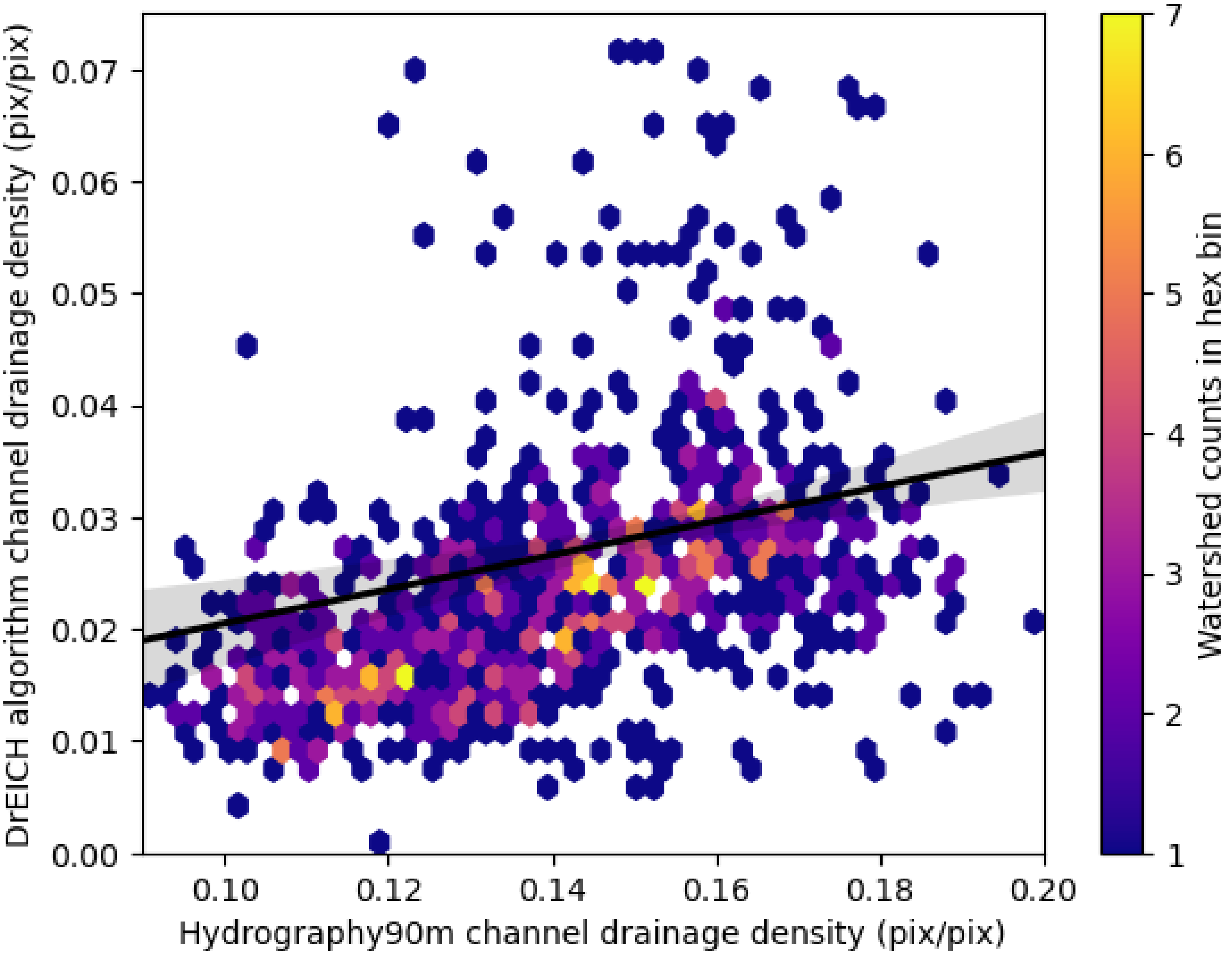

Fig. S7. Comparison of drainage density calculated the two sources of channel data (Hydrography90m (4) and DrEICH algorithm (5, 6))

Joanmarie Del Vecchio, Marisa Palucis, Colin Meyer

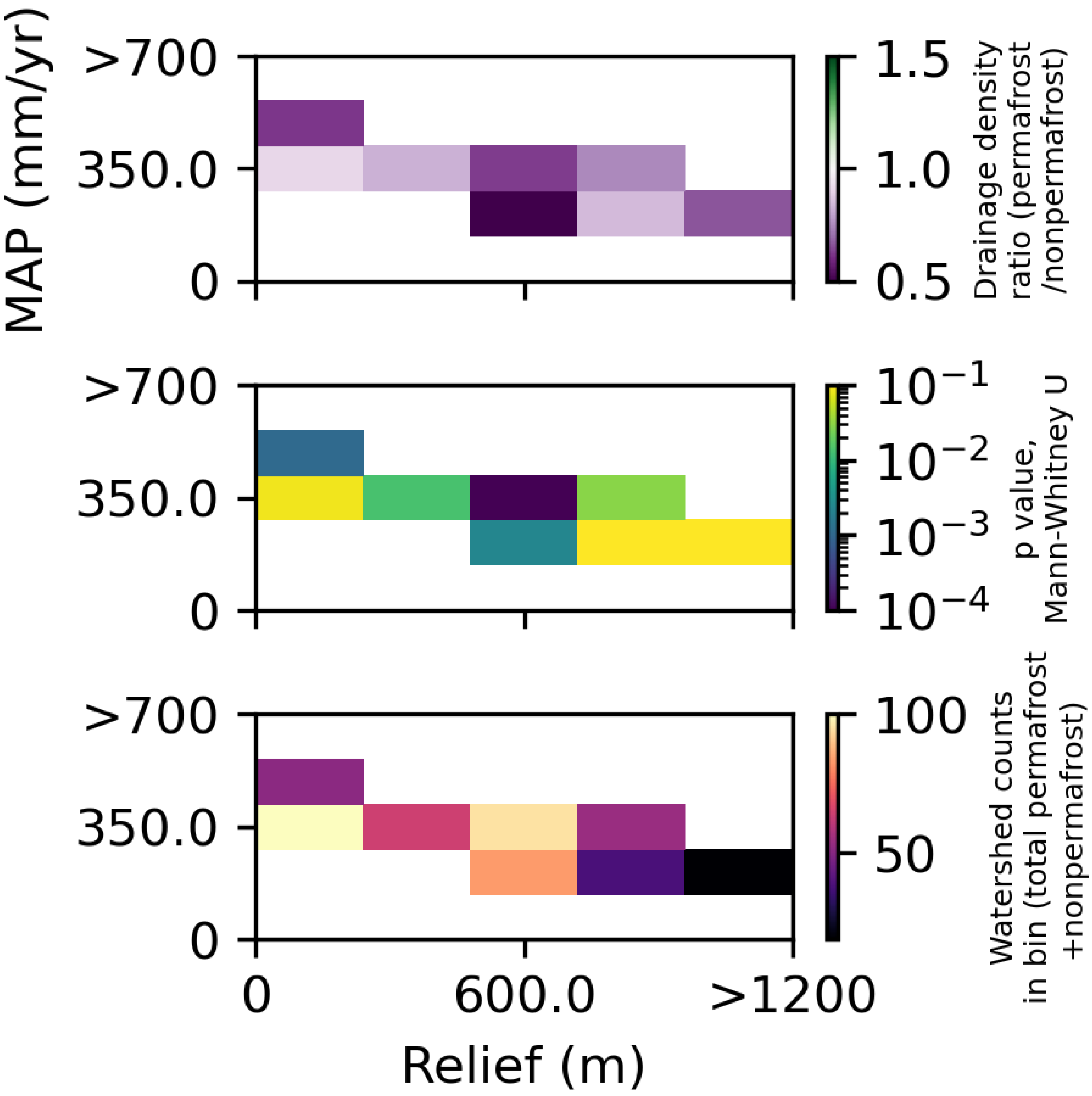

Fig. S8. Of the 936 subset watersheds from continuous permafrost and nonpermafrost, 504 had significant overlap in MAP-relief space and are compared here.

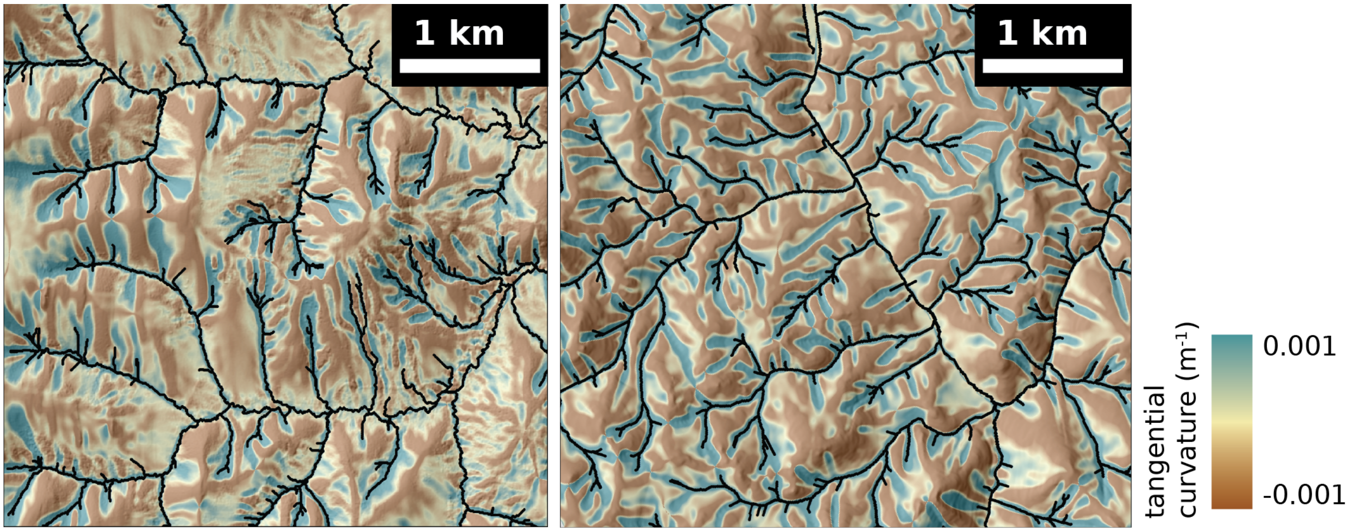

HYBAS ID: 3100413670
Latitude: 60.3
Longitude: 137.33
Mean annual temperature: -11.1 C
Relief: 705 m
Mean annual precipitation: 417 mm
Hydrography90m DD: 0.101
DrEICH DD: 0.017

HYBAS ID: 7100377840
Latitude: 46.17
Longitude: -112.46
Mean annual temperature: 2.3 C
Relief: 612 m
Mean annual precipitation: 436 mm
Hydrography90m DD: 0.137
DrEICH DD: 0.021

Fig. S9. Randomly selected watershed from continuous permafrost and non-permafrost landscapes (same as Figure 1A and 1D) with tangential curvature mapped with a hillshade and the DrEICH algorithm-generated channel networks.

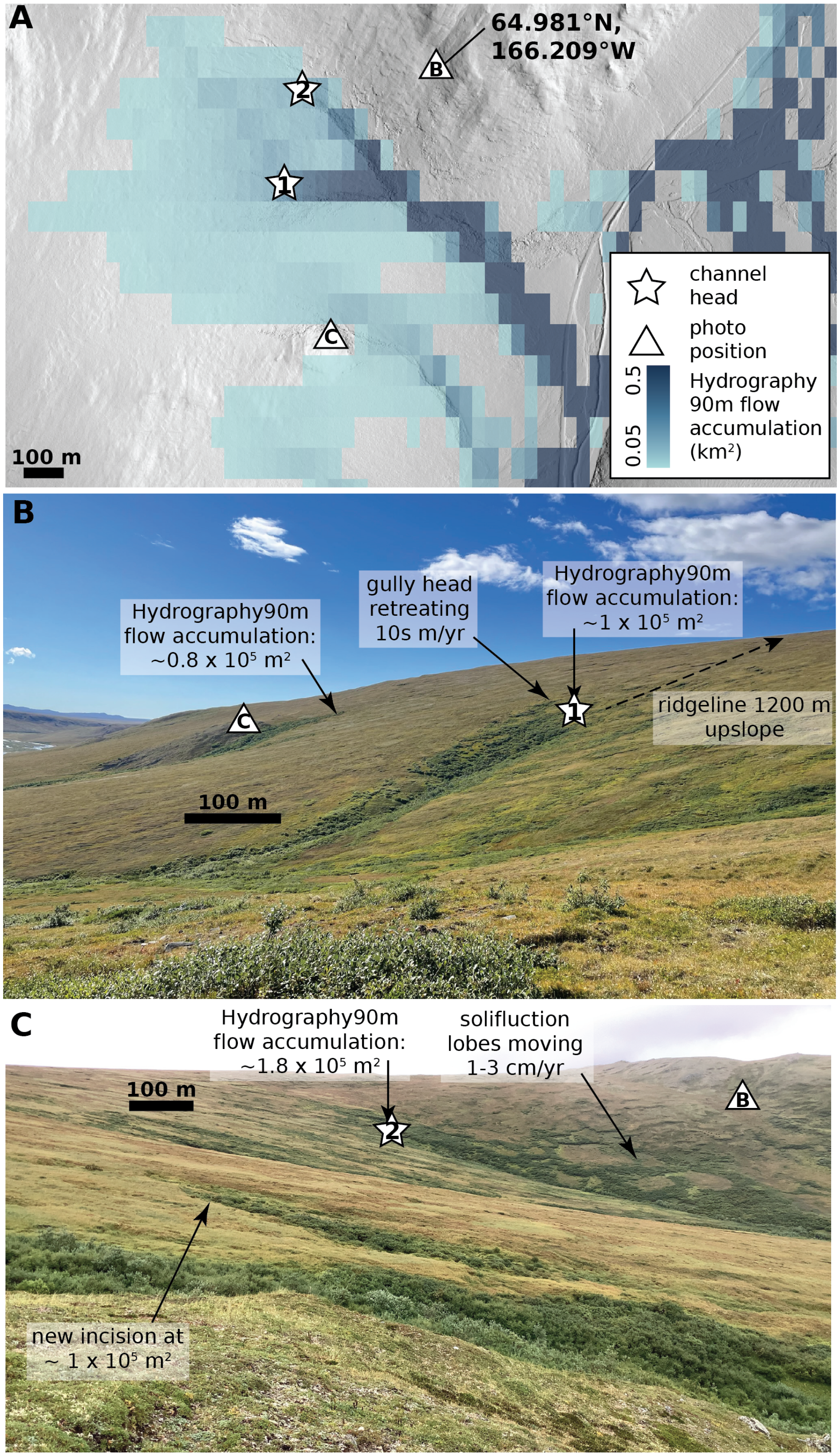

Fig. S10. Field images of permafrost channel heads and corresponding Hydrography90m data. (A) Lidar hillshade of a series of watersheds in western Alaska, with Hydrography90m flow accumulation data overlain. Numbered stars are channel heads pictrured in (B) and (C). Lettered triangles are photo views of their respective panels. (B) View with channel head 1, noting a nearby gully retreating quickly upslope as described in (7). (C) A view of channel head 2 with solifluction lobes in the background, with movement rates described in (7). Lidar elevation data available at https://ngee.ornl.gov/ngeedata/NGA270/data/Teller_MM47

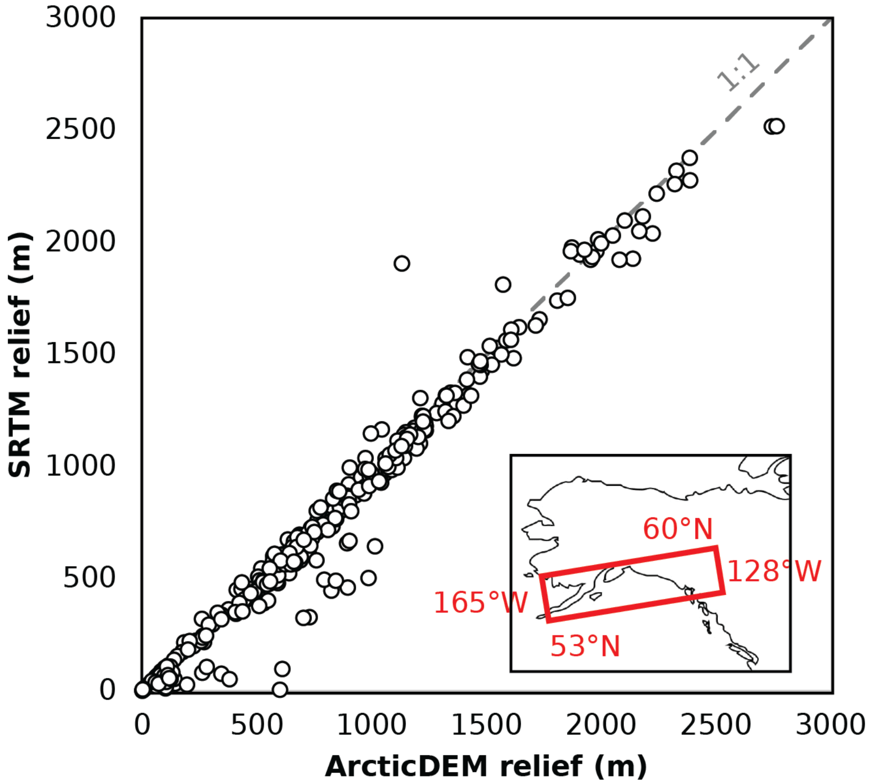

Fig. S11. Comparison between watershed relief as measured on the SRTM DEM and the ArcticDEM for watersheds that contain toporgraphic data from both elevation models in southern Alaska and western Canada.

Table S1. Number of watersheds grouped by both permafrost extent and glacial history categories

Permafrost extent	Glacial history	Watershed counts
Continuous	LGM	2004
	MIS6	4549
	Not glaciated	7923
Discontinuous	LGM	1911
	MIS6	1321
	Not glaciated	2772
Sporadic	LGM	2784
	MIS6	977
	Not glaciated	2104
Isolated	LGM	2177
	MIS6	338
	Not glaciated	3204
No permafrost	LGM	10645
	MIS6	3755
	Not glaciated	23220

Table S2. Parameters* for DEM preprocessing, curvature, and channel head algorithms for the LSDTopoTools (8) channel extraction algorithms

Parameter	value
`carve_before_fill`	True
`raster_is_filled`	False
`surface_fitting_radius` (m)	100
`threshold_contributing_pixels` (pixels)	5000
`A_0`	1.0
`m_over_n`	0.5
`pruning_drainage_area`	10
`connected_components_threshold`	10

*all other omitted parameters were set to defaults

References

1. J Brown, KM Hinkel, FE Nelson, The circumpolar active layer monitoring (calm) program: Research designs and initial results. *Polar Geogr.* **24**, 166–258 (2000) Publisher: Taylor & Francis _eprint: https://doi.org/10.1080/10889370009377698.
2. CL Batchelor, et al., The configuration of Northern Hemisphere ice sheets through the Quaternary. *Nat. Commun.* **10**, 1–10 (2019) Publisher: Springer US.
3. RJ Hijmans, SE Cameron, JL Parra, PG Jones, A Jarvis, Very high resolution interpolated climate surfaces for global land areas. *Int. J. Climatol. A J. Royal Meteorol. Soc.* **25**, 1965–1978 (2005).
4. G Amatulli, et al., Hydrography90m: a new high-resolution global hydrographic dataset. *Earth Syst. Sci. Data* **14**, 4525–4550 (2022) Publisher: Copernicus GmbH.
5. SM Mudd, et al., LSDTopoTools2 (2021).
6. FJ Clubb, SM Mudd, DT Milodowski, MD Hurst, LJ Slater, Objective extraction of channel heads from high-resolution topographic data. *Water Resour. Res.* pp. 5375–5377 (2014) arXiv: 10.1002/2014WR016527 ISBN: 6176273099.
7. J Del Vecchio, et al., Patterns and rates of soil movement and shallow failures across several small watersheds on the Seward Peninsula, Alaska. *Earth Surf. Dyn. Discuss.* pp. 1–28 (2023) Publisher: Copernicus GmbH.
8. SM Mudd, et al., LSDTopoTools2 (2022).
